# Optimal SARS-CoV-2 vaccine allocation using real-time attack-rate estimates in Rhode Island and Massachusetts

**DOI:** 10.1186/s12916-021-02038-w

**Published:** 2021-07-13

**Authors:** Thu Nguyen-Anh Tran, Nathan B. Wikle, Emmy Albert, Haider Inam, Emily Strong, Karel Brinda, Scott M. Leighow, Fuhan Yang, Sajid Hossain, Justin R. Pritchard, Philip Chan, William P. Hanage, Ephraim M. Hanks, Maciej F. Boni

**Affiliations:** 1 Center for Infectious Disease Dynamics, Department of Biology, Pennsylvania State University, University Park, PA USA; 2 Center for Infectious Disease Dynamics, Department of Statistics, Pennsylvania State University, University Park, PA USA; 3 Department of Physics, Pennsylvania State University, University Park, PA USA; 4 Center for Infectious Disease Dynamics, Department of Bioengineering, Pennsylvania State University, University Park, PA USA; 5 Center for Communicable Disease Dynamics, Department of Epidemiology, Harvard T. H. Chan School of Public Health, Boston, MA USA; 6 Department of Biomedical Informatics, Harvard Medical School, Boston, MA USA; 7 Yale School of Medicine, Yale University, New Haven, CT USA; 8 Department of Medicine, Brown University, Providence, RI USA

**Keywords:** SARS-CoV-2, Vaccination, Optimal vaccine allocation, Mathematical modeling, Real-time seroprevalence

## Abstract

**Background:**

When three SARS-CoV-2 vaccines came to market in Europe and North America in the winter of 2020–2021, distribution networks were in a race against a major epidemiological wave of SARS-CoV-2 that began in autumn 2020. Rapid and optimized vaccine allocation was critical during this time. With 95% efficacy reported for two of the vaccines, near-term public health needs likely require that distribution is prioritized to the elderly, health care workers, teachers, essential workers, and individuals with comorbidities putting them at risk of severe clinical progression.

**Methods:**

We evaluate various age-based vaccine distributions using a validated mathematical model based on current epidemic trends in Rhode Island and Massachusetts. We allow for varying waning efficacy of vaccine-induced immunity, as this has not yet been measured. We account for the fact that known COVID-positive cases may not have been included in the first round of vaccination. And, we account for age-specific immune patterns in both states at the time of the start of the vaccination program. Our analysis assumes that health systems during winter 2020–2021 had equal staffing and capacity to previous phases of the SARS-CoV-2 epidemic; we do not consider the effects of understaffed hospitals or unvaccinated medical staff.

**Results:**

We find that allocating a substantial proportion (>75*%*) of vaccine supply to individuals over the age of 70 is optimal in terms of reducing total cumulative deaths through mid-2021. This result is robust to different profiles of waning vaccine efficacy and several different assumptions on age mixing during and after lockdown periods. As we do not explicitly model other high-mortality groups, our results on vaccine allocation apply to all groups at high risk of mortality if infected. A median of 327 to 340 deaths can be avoided in Rhode Island (3444 to 3647 in Massachusetts) by optimizing vaccine allocation and vaccinating the elderly first. The vaccination campaigns are expected to save a median of 639 to 664 lives in Rhode Island and 6278 to 6618 lives in Massachusetts in the first half of 2021 when compared to a scenario with no vaccine. A policy of vaccinating only seronegative individuals avoids redundancy in vaccine use on individuals that may already be immune, and would result in 0.5% to 1% reductions in cumulative hospitalizations and deaths by mid-2021.

**Conclusions:**

Assuming high vaccination coverage (>28*%*) and no major changes in distancing, masking, gathering size, hygiene guidelines, and virus transmissibility between 1 January 2021 and 1 July 2021 a combination of vaccination and population immunity may lead to low or near-zero transmission levels by the second quarter of 2021.

**Supplementary Information:**

The online version contains supplementary material available at (10.1186/s12916-021-02038-w).

## Background

The international effort to bring a SARS-CoV-2 vaccine to market began in January 2020 with the release of the viral genome sequence [1]. Development of two mRNA vaccines and dozens of other vaccine candidates began shortly thereafter [2, 3], and two vaccine candidates (Moderna and Pfizer/BioNTech) were approved for use in the USA by the US Food and Drug Administration in December 2020 [4, 5]. A third vaccine was approved in February 2021 [6]. Rollout of these two vaccines in the USA was the best near-term hope of stopping the epidemic by spring/summer 2021 and keeping the total death toll in the USA below half a million. The Centers for Disease Control and Prevention [7–9] together with state-level departments of health [10, 11] developed vaccine distribution and prioritization plans for the first batch of doses and the first few months of distribution.

In vaccination campaigns, prioritization of certain population groups over others can influence the success of the campaign. The classic trade-off in vaccine distribution programs is between vaccinating high-contact versus high-risk individuals [12–15], and the optimal approach depends on (1) the outcome measure being used—e.g., case numbers, hospitalizations, or deaths, (2) the vaccine supply, (3) the mortality rate in the at-risk age groups, and (4) the current level of transmission. For long-term planning in influenza vaccination, there is empirical evidence that vaccination of children (the high-contact group) can lead to reduced case numbers, morbidity, and deaths for all age groups [16]; however, short-term planning is much more sensitive to small changes in rollout details and the current state of the epidemic. The benefit of reduced transmission may come at a delay from the start of the vaccination campaign, and thus it is often safer to protect vulnerable groups with the direct benefit of vaccination rather than the indirect benefits of vaccinating others. This is especially true for the COVID-19 pandemic as SARS-CoV-2 infections have an infection mortality rate that is >10 times higher than that of influenza virus [17].

Vaccinating populations with real-time information on seroprevalence or attack rate—used interchangeably here as the seroprevalence is simply a 3-week delayed version of the attack rate—allows a public health system to introduce efficiencies into vaccine allocation. Here, we evaluate different age-distributions for vaccine rollout given current age-stratified attack rate estimates in Rhode Island and Massachusetts [18]. While we assume that confirmed seropositive individuals (i.e., those who were confirmed COVID-19 cases in the past) will not receive the vaccine in the first rounds of vaccine distribution, this will understandably be implemented through voluntary compliance as it will not be possible to have a “seronegatives only” vaccine program in place due to the rapid schedule of shipping, deployment, staffing, and vaccination. The current seroprevalence also factors into allocation decisions as the pace of vaccination and simultaneous transmission will determine how quickly the population approaches the herd-immunity threshold, an approach that has beneficial non-linear effects in reducing the final tally of infected individuals [19–21].

Using a mathematical model whose fit to Rhode Island and Massachusetts COVID-19 data was described in Wikle et al. [18], we consider seven different vaccine efficacy profiles/halflives (currently an unknown), we evaluate the individual importance of each 10-year age band to vaccination outcomes, we compare several common age allocations under assumptions of high and low vaccine supply, and we evaluate the magnitude of population-level effects if vaccination is dependent on serostatus. We assume that health care workers and front-line medical staff are vaccinated first, as our model is not able to evaluate the effects of a stressed and understaffed health system.

## Methods

### Model and fitting

We use a mathematical model developed by Wikle et al. [18] and fit to Rhode Island and Massachusetts data so that vaccination campaigns can be evaluated in the context of the number of individuals already infected at the time the vaccination campaign began. As of 30 November 2020 total estimated attack rates were 20.7*%* (95% CredInt: 17.3–24.1%) in Rhode Island and 12.5*%* (95% CredInt: 11.5–13.5%) in Massachusetts [22]. We upgraded the model parameterization by using age-contact matrices measured in Belgium (BE), as part of the CoMix study, to describe lockdown and post-lockdown mixing patterns [23]. The eight age bands in the CoMix data [23] were transformed into our nine age bands using the socialmixr R package [24]. To check the robustness of our conclusions to different mixing assumptions, we also parameterized the model using contact patterns measured in United Kingdom (UK) in March 2020 [25]. Since the UK contact survey did not enroll participants aged below 18, we assumed age group 0–9 and 10–19 had symmetric contact patterns. The oldest age group in the UK contact survey was 70+ while that in our model was 80+; therefore, when adopting results from the UK survey, we assumed the 70–79 and 80+ age groups had identical contact patterns. We conducted a third analysis where parameterization was done with a combination of UK and BE contact matrices to represent contact patterns during (UK matrix) and after (BE matrix) lockdown. Results from the analysis using only BE contact patterns are presented in the main text and Figure S1-S29. Results from analyses involving UK contact patterns are shown in the Supplementary Materials as Figure S30-S40.

All simulation results are presented as medians. We draw ten sets of parameters from posterior distributions of the new model fit, and we present simulation results as the median value of these ten simulations. The new model’s median 30 November 2020 attack rates ares 19.6% for RI and 12.9% for MA.

**Fig. 1:**
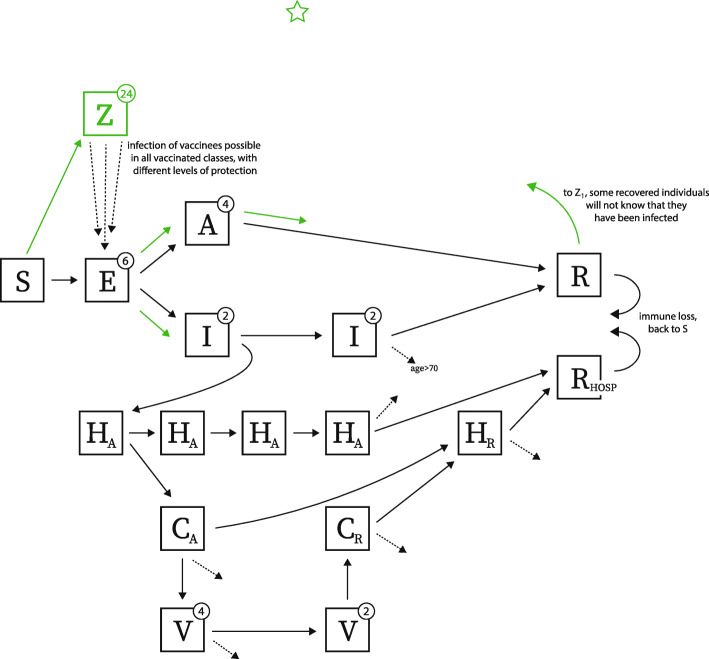
We extend the model from [18] to include a 24-stage vaccinated compartment, denoted by *Z*. Other compartments are susceptible (*S*), exposed (*E*), asymptomatic (*A*), infected and symptomatic but not hospitalized (*I*), hospitalized in acute stage of infection (*H*_*A*_), in critical care (acute stage) (*C*_*A*_), on mechanical ventilation (*V*), in critical care recovering phase (*C*_*R*_), hospitalized and recovering (*H*_*R*_), recovered (*R*), recovered from hospitalization (*R*_HOSP_). Green arrows show progression after an individual is vaccinated; dashed arrows indicate death. Exposed and asymptomatic individuals who are vaccinated receive no benefit from the vaccine and progress on their normal infection/disease course. We assume vaccines are not given to individuals with past confirmed infection. However, if no serological test is performed before vaccinating, it is possible that individuals in exposed (*E*), asymptomatic (*A*), and recovered (*R*) classes will have an equal chance of receiving the vaccine as truly susceptible individuals. Re-infection is possible as shown by arrows going from recovered *R* and *R*_HOSP_ back to susceptible compartment *S*. Vaccinees in the *Z* class are not fully protected from infection since we allow waning of vaccine-induced immunity over time; infection of vaccinated individuals is shows with the dashed arrows from *Z* to *E*

**Fig. 2:**
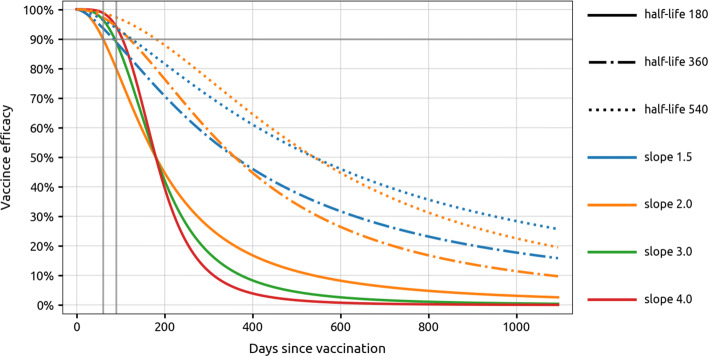
We assume that vaccine efficacy wanes over time following a Hill-like function characterized by an efficacy half-life and slope (Equation 1). The half-life (as shown with different line styles) denotes on which day after vaccination the efficacy drops below the 50% mark. The slope (shown with different line colors) denotes how fast the vaccine loses its efficacy; for example, considering the solid lines in this figure (half-life = 180), efficacy of the orange vaccine (slope = 2) reduces from 70 to 50% in 64 days while that of the red vaccine (slope = 4) does so in 36 days. Here, we choose 7 vaccine profiles whose 60-day and/or 90-day efficacies are at least 90% to represent the currently approved vaccines from Pfizer/BioNTech and Moderna

**Fig. 3:**
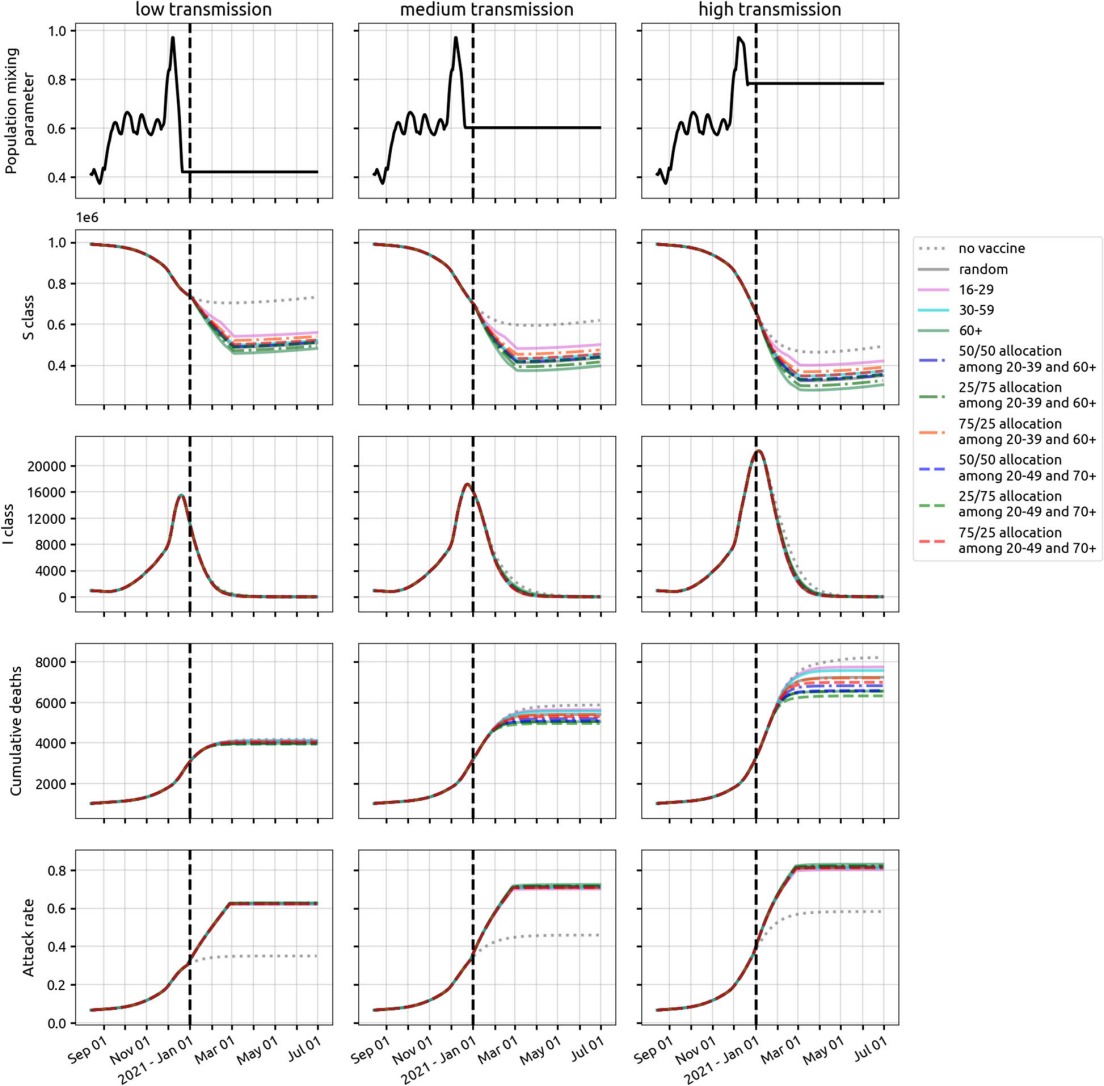
Dynamics of SARS-CoV-2 epidemic in Rhode Island under three different transmission scenarios from 1 December 2020 onwards. The vaccine profile shown here has efficacy half-life of 180 days and slope of 2. The vaccination campaign covers 300,000 people (28.3*%* population coverage) and ends on 4 March 2021. Under medium transmission (middle column), the population mixing parameter is assumed to remain at the mean mixing level from September through November 2020. This mixing parameter (top row) is reduced by 30% and increased by 30% to achieve low and high transmission settings, respectively. The second and third row show the size of susceptible (*S*) and symptomatic (*I*) compartment over time. The fourth row shows the cumulative deaths, both at home and in hospitals. The last row shows seroprevalence (including vaccinees) in Rhode Island from 15 August 2020 to 30 June 2021. With no vaccination (dotted gray line), attack rate would reach 34.9*%*,45.9*%*, and 58.2*%* by 30 June 2021 under low, medium, and high transmission settings, respectively. Scenarios and dynamics for Massachusetts shown in Additional file 1: Figure S14

To model vaccination with waning vaccine efficacy, we add a 24-stage vaccinated state (classes *Z*_1_ to *Z*_24_ in Fig. 1) in order to allow vaccine efficacy to be modeled as *x*% efficacy at *n* weeks post-vaccination. Clearly, 24 vaccine efficacy estimates for different time points have not been published, but we use this 24-compartment chain to model the expected gradual changes in vaccine efficacy from the early and measured stages in clinical trials (60 to 90 days after vaccination, with observed efficacy around 95%) to stages 12 or 18 months later when the vaccine is assumed to have an exponentially waned efficacy (several parameterizations are explored). Even for early-stage efficacy, these data are not yet available because we do not know the average enrollment duration of patients in the two key trials. Nevertheless, we make several reasonable choices for shapes of this efficacy function based on the enrollment dates of each trial [26, 27]. The efficacy curves in Fig. 2 are parameterized with a Hill-like function, HL^*s*^/(HL^*s*^+*T*^*s*^), where HL is the efficacy half-life, *s* is the slope, and *T* is the time post-vaccination. To translate this individual efficacy into a population-level model, we define the vaccine efficacy (VE_*j*_) in the vaccinated class *Z*_*j*_ as

1 $$ \text{VE}_{j}(t_j) \;\;=\;\; \frac{(1 - e^{-\text{FOI} \cdot t_{j}}) \;-\; (1 - e^{-a_{j} \cdot \text{FOI} \cdot t_{j}})}{1 - e^{-\text{FOI} \cdot t_{j}}}  \;,  $$

where *t*_*j*_ is the time after the final vaccine dose, FOI is the force of infection in the population, and *a*_*j*_ is the relative reduction in susceptibility for a vaccinated individual in class *Z*_*j*_ (which is *t*_*j*_ days after the final dose of vaccination). We assume an FOI of 0.001 per day, and for *t*_*j*_=60 or *t*_*j*_=90 days we assume 95% efficacy and solve for *a*_*j*_. For other values of *t*_*j*_, the vaccine efficacy is assumed to follow one of the seven patterns in Fig. 2, and 24 *a*_*j*_ values (relative risk parameters for a fixed time point after vaccination) are solved for. We assume the force of infection from 1 December 2021 onward is constant and we explore three transmission scenarios. In the low-transmission scenario, transmission levels in Rhode Island and Massachusetts revert to their late spring and summer levels. Under medium transmission, transmission levels are set to their September through November mean value. Under high transmission, wintertime transmission levels continue through spring 2021; see Fig. 3. The medium transmission scenario is explored in the main text, and figures for the high and low scenarios are included in the Supplementary Materials.

Individuals can be vaccinated if they are in the susceptible class. Individuals who experienced asymptomatic infection and are in the recovered class can also be vaccinated, according to whether they were likely to have been asymptomatic or not [28] and confirmed PCR-positive or not (reporting parameter *ρ* in Wikle et al. [18]). Individuals in the exposed class *E* or the asymptomatic class *A* can be chosen for vaccination but we assume that they progress through their normal course of infection with no effect of the vaccine, as vaccination will have occurred for these individuals at a time when their immune system has already been exposed to whole live virus SARS-CoV-2.

Vaccinated individuals from *S* and *R* are moved into the vaccinated class *Z*_1_. This means that we assume all vaccinated individuals become seropositive after vaccination. These individuals can then be infected, despite their seropositivity, at the relative rate *a*_*j*_ in the class *Z*_*j*_, solved for in equation (1). Individuals progress from *Z*_1_ through to *Z*_24_ and back to *S* over the course of 540 days. Likewise, individuals entering the recovered class *R* are all assumed to be seropositive, with naturally immunity waning after a mean 540 days.

### Vaccination strategies

Vaccination strategies are considered where doses are made available for 4.7*%* or 28.3*%* of the population. We call these the low and high supply scenarios: 50,000 or 300,000 vaccinations available in RI, and 300,000 or 1.8 million vaccinations available in MA. Distribution lasts from 14 December 2020 through 13 January 2021 (4.7*%* vaccine coverage) or 14 December 2020 to 4 March 2021 (28.3*%* vaccine coverage).

The following age-based strategies are considered: (1) random, where any individual ≥16 in the population can be chosen for vaccination on a particular day; (2) 16–29 age group only; (3) 30–59 age group only; (4) 60-and-above age group only; vaccine supply is allocated to the 20–39 and 60+ age groups in proportions of (5) 75/25, (6) 50/50, (7) 25/75; vaccine supply is allocated to the 20–49 and 70+ age groups in proportions of (8) 75/25, (9) 50/50, and (10) 25/75. If there is sufficient supply to cover an entire age group, the remaining vaccines are allocated to the second age group. If both age groups have been covered completely, the remaining vaccines are distributed at random in the population to all individuals over the age of 16.

In addition, using the nine 10-year age bands in our model, we consider all 2^9^−1=511 possible combinations of age bands in the vaccination strategy. We take this approach to see if including or excluding a particular age band has a large effect on the results, after marginalizing over the inclusion/exclusion of the other age groups. As evaluation criteria, we consider the median total cumulative number of cases, hospitalizations, and deaths through to 30 June 2021, where the median is taken over ten forward simulations using ten parameter samples from the posterior of the model fit. Our model does not evaluate the benefits of vaccinating health care workers. We assume that health care and front-line medical staff are vaccinated first, and that hospital capacity and staffing during the winter wave are not affected by absenteeism or a surge of COVID-19 cases.

## Results

As the duration of immunity from the two current high-efficacy vaccines (Pfizer/BioNTech and Moderna) to have completed phase 3 clinical trials is not known, we build several vaccine profiles that have 95% vaccine efficacy at 60 to 90 days post-vaccination, with four different slopes of waning vaccine efficacy (Fig. 2). In the most pessimistic scenario considered here, vaccine efficacy wanes to 50% after 6 months and continues to lower levels via an exponential decay thereafter (three solid lines Fig. 2). Seven profiles are considered in all, and while there are quantitative differences in the population-level outcomes when considering different vaccine profiles, qualitatively the 6-month outcomes on age prioritization (see below) are not sensitive to the exact shape of the vaccine efficacy profile. Outcomes after 12 months are sensitive to the vaccine profile, with shorter half-life vaccines likely requiring booster campaigns to be planned for 2022 (results not shown).

**Fig. 4:**
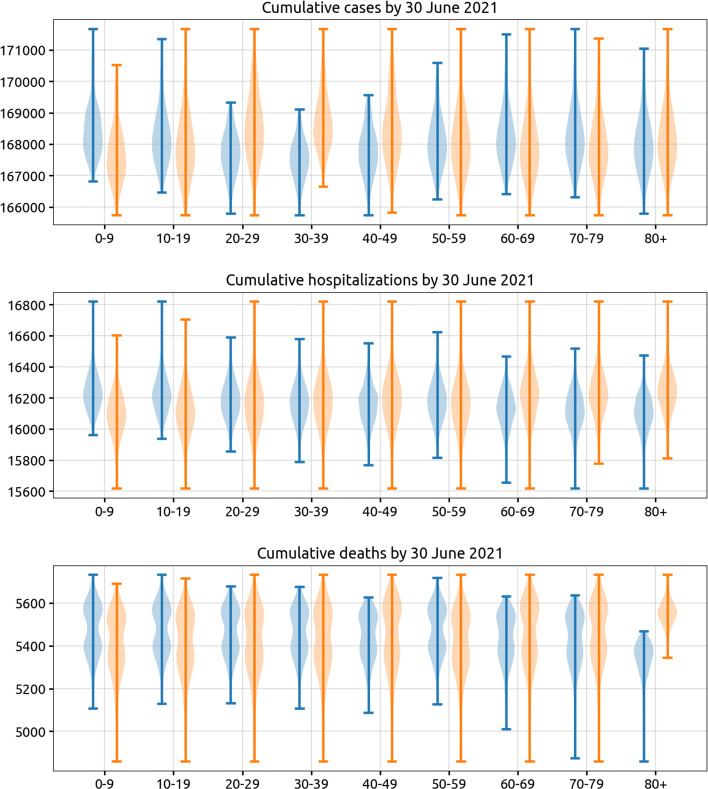
Impact of including (blue) or excluding (orange) each age group in a vaccination policy, measured as reductions in cumulative cases, hospitalizations, and deaths in Rhode Island by 30 June 2021. With nine age classes in the model, there are 2^9^−1=511 possible age-based vaccination strategies where the vaccine supply is equally distributed among the participating age groups. The vaccination campaign in this figure ends on 4 March 2021 and the total vaccine supply is enough to vaccinate 300,000 people (28.3% coverage). Vaccine efficacy half-life here is 360 with slope = 2. Each violin plot shows the distribution across 256 (blue) or 255 (orange) strategies which include or exclude the corresponding age group. Each strategy was evaluated by taking the median of ten runs with ten different sets of parameters drawn from the inference [18] posterior distributions. Campaigns which cover the 30–39 age group would reduce the median of cumulative cases by 0.67*%* and cumulative hospitalizations by 0.06*%* compared to those not covering this age group. The median of cumulative deaths drops by 3.58*%* when targeting the 80+ age group when compared to strategies that do not include the 80+ age group. Results for Massachusetts shown in Additional file 1: Figure S13

The most straightforward approach to maximizing public health utility out of every vaccine dose is to vaccinate either high-contact and high-risk individuals. It is known that contact rates vary across the age groups, and that for SARS-CoV-2 risk and clinical severity increase monotonically with increasing age. To determine the average sizes of the effects of including or excluding certain age groups for SARS-CoV-2 vaccination in Rhode Island and Massachusetts, we considered all possible strategies (511 in total) defined by inclusion/exclusion based on 10-year age band. We assumed that transmission from 1 December 2020 onwards would persist at the mean level observed in September - November 2020 (the “medium transmission” scenario). The violin plots in Fig. 4 show the total number of cases, hospitalizations, and deaths when a particular age group is included (blue) or excluded (orange) in a vaccination strategy. Each of the vaccination strategies considered here simply has random distribution among individuals in the included age groups. Including the middle age groups (20–39 or 20–49) results in an overall benefit in reducing case numbers, with average reductions of 0.42% (IQR: 0.29–0.66%) when including the 20–29 age group, 0.67% (IQR: 0.51–0.89%) for the 30–39 age group, and 0.35% (IQR: 0.24–0.54%) for the 40-49 age group. When evaluating hospitalizations, including the 70-79 age group results in 0.63% (IQR: 0.50–0.83%) fewer hospitalizations, and including the 80+ age group results in 0.84% (IQR: 0.68–1.09%) fewer hospitalizations. When evaluating deaths as the relevant outcome measure, including the 70–79 age group results in 0.48% (IQR: 0.31–0.79%) fewer deaths, and including the 80+ age group results in 3.58% (IQR: 2.98–4.63%) fewer deaths. As expected, the high-risk (70+) and high-contact (20–49) age groups should be priority targets for vaccination campaigns, with an elderly focused campaign being the simplest approach to minimizing fatalities in the short-term.

**Fig. 5:**
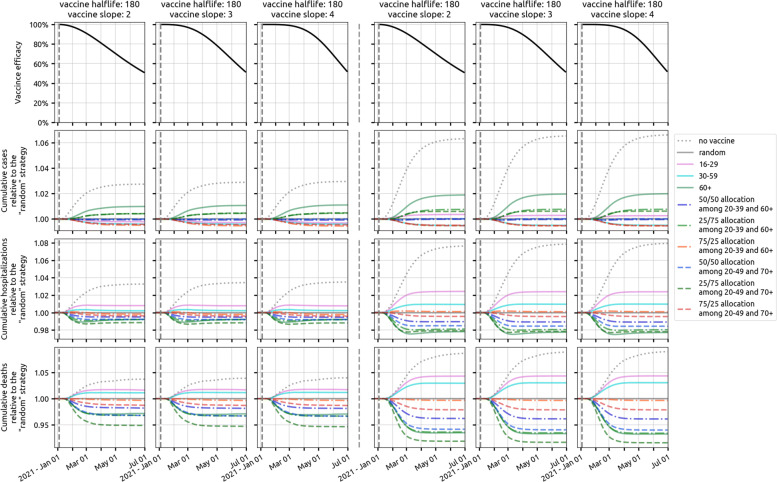
Comparison of ten vaccination strategies in Rhode Island based on cumulative cases (second row), hospitalizations (third row), and deaths (fourth row), given two levels of vaccine supply and under the three most pessimistic assumptions for vaccine profile (top row). The left three columns show outcomes when the vaccine supply is low (50,000 vaccines), and the right three columns show outcomes for high vaccine supply (300,000 vaccines). Our reference strategy is “random” (solid gray line) where everybody has an equal chance of getting vaccinated. The solid pink, cyan, and green lines are strategies which cover the 16–29, 30–59, and 60+ age groups, respectively. The dashed-dotted lines are strategies targeting 20–39 and 60+ age groups with different distribution of vaccine supply to the two age groups (e.g., blue is 25% of the allocation for 20–39 and 75% of the allocation for 60+). The dashed lines are strategies which include 20–49 and 70+ age groups, which are the key age groups identified in Fig. 4. Results for medium transmission scenario in Massachusetts shown in Additional file 1: Figure S20

**Fig. 6:**
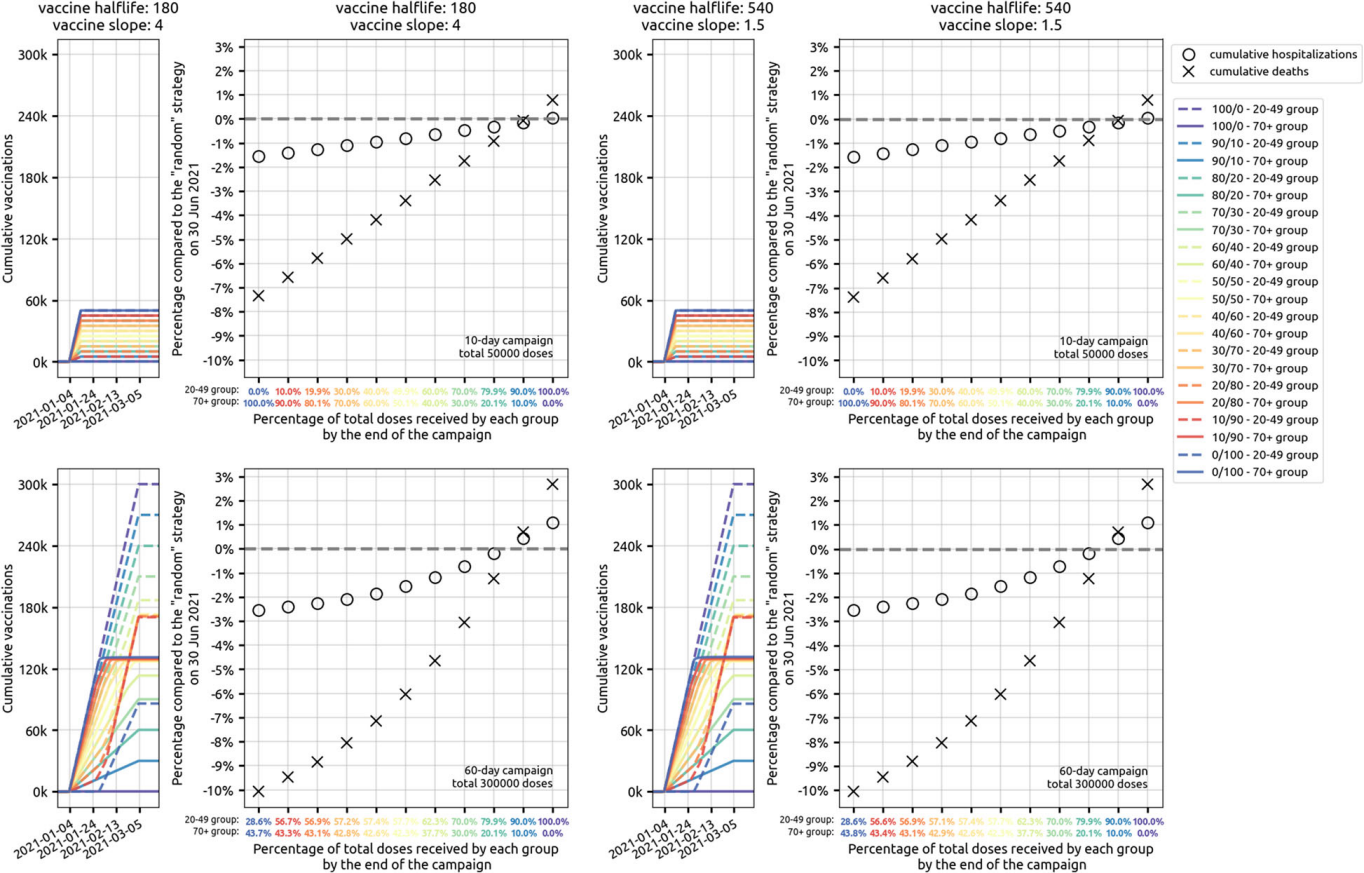
Analysis of different age allocations for strategies focused on the 20–49 and 70+ age groups, ranging from a 10/90 allocation (90% of vaccines initially given to 70+ age group) to a 90/10 allocation (90% of vaccines initially given to 20–49 age group). Top row shows the low-supply scenario for Rhode Island (50,000 vaccines) and bottom row shows the high supply scenario (300,000 vaccines available). Left and right columns show scenarios for different vaccine profiles, with a 180-day half-life vaccine shown on the left and a 540-day half-life vaccine on the right. Narrow plots show the cumulative vaccinations by age class, for the 20–49 (dashed lines) and 70+ (solid lines) groups under different allocation strategies. Circles and crosses show the percentage reduction in hospitalizations (circles) and deaths (crosses) when compared to a random distribution strategy. The *x*-axis shows the final vaccine allocation to the 20–49 and 70+ age groups. In the top row (low supply), these allocations are 10/90, 20/80 etc., as planned. But, in the bottom row, for 10/90, 20/80,..., 50/50, allocation, there is nearly enough vaccine for all individuals in these two age classes, and when one age group is fully vaccinated the remainder of the supply is used for the second age group. Thus, while the final age distribution does not vary much 57/43 to 58/42 under amply vaccine supply, it does affect which groups get vaccinated earlier. Under ample vaccine supply, a 10/90 allocation means that older individuals get vaccinated earlier and more deaths and hospitalizations are averted. For 0/100 and 100/0 allocation, after the target group is fully covered, the remaining vaccine doses are distributed randomly to other age groups in the population. Results for medium transmission scenario in Massachusetts shown in Additional file 1: Figure S23

In a low-supply scenario with 50,000 vaccinations available in Rhode Island (4.7*%* coverage) during the initial rollout, allocating 25% of vaccines to the 20–49 age group and the remaining 75% to the 70+ age group is optimal (among the strategies evaluated) in terms of minimizing deaths and hospitalizations; see left three columns, Fig. 5. While the two different 25/75 (younger/older) allocations we evaluated are optimal for death and hospitalization outcomes, they are associated with the highest final case counts meaning that they have the smallest effect on overall transmission reduction. The 75/25 allocations are best at reducing case counts and near-optimal at reducing hospitalizations (red lines, Fig. 5). But, as the 75/25 allocations are majority focused on the younger age classes, they are associated with a substantially higher final death count by mid-2021 as they fail to provide enough vaccination for the age groups with the highest risk of dying if infected. Figure 6 (top row) shows that prioritization of vaccine allocation to the 70+ age group has a modest effect on reducing hospitalizations and a substantial effect on reducing deaths, as much as a 7% difference in cumulative deaths through 30 June 2021, when viewing the two extreme strategies (10/90 versus 90/10 allocations). This corresponds to more than a hundred deaths in RI and hundreds of deaths in Massachusetts. Because the vaccine supply is low absolute benefits are also low, and most age-allocation strategies are associated with case/hospitalization/death outcomes that are within 5% of a simple random allocation strategy.

In a more optimistic scenario—300,000 vaccines procured in Rhode Island (28.3*%* population coverage)—strategies focused on the elderly can reduce cumulative death numbers by as much as 10% when compared to a random distribution strategy (three right columns, Fig. 5). The optimal strategy among those evaluated is a 25/75 distribution to the 20–49 and 70+ age groups, outperforming the 60+ strategy and the 20–39 and 60+ strategy with 25/75 allocation, both of which vaccinate more elderly individuals but have sub-optimal outcomes because they have too small of an effect on transmission reduction. With ample vaccine supply, most individuals in the 20–49 and 70+ age groups will be vaccinated. However, the allocation is still important in deciding which groups are vaccinated earlier than others. Figure 6 (bottom row) shows that the percentage of the entire allocation going to the 20–49 and 70+ age groups does not differ greatly between a starting allocation of 90/10 and 10/90 (because everyone is eventually vaccinated anyway), but in a 10/90 allocation (i.e., 90% of vaccines reserved for 70+) the older age groups receive the vaccine earlier. Under a 10/90 allocation, the mortality benefits are substantial, with 11% fewer deaths when compared to a 90/10 allocation in which the 20–49 age group is vaccinated earlier. For both low and high supply scenarios, strategies focusing predominantly on higher-contact age groups—e.g., strategies where 50% or more of the vaccine supply is allocated to younger age groups—are always sub-optimal at minimizing deaths.

The high and low vaccine supply scenarios have identical implications for the current epidemiological situation in Massachusetts. Figure S20 shows that the 25/75 vaccine allocation to the 20–49 and 70+ age groups is also the optimal allocation (when considering deaths as the primary outcome) among those examined for Massachusetts. Under a scenario of low vaccine supply, deaths are reduced by approximately 8% when comparing to a strategy of random vaccine allocation; under high vaccine supply, deaths are reduced by approximately 13% when comparing to a random strategy. As in Rhode Island, prioritization of the 70+ group (or any group with a similar mortality risk) leads to optimal outcomes (see Figure S23).

For Rhode Island, assuming a total of 300,000 vaccinations (28.3*%* coverage), a strategy that is targeted 25/75 at the 20–49 and 70+ age groups will results in 335–353 (from the least to the most optimistic vaccine efficacy profile) fewer hospitalizations and 327–340 fewer deaths by 30 June 2021 than a random allocation strategy. Compared to the unmitigated scenario (no vaccine), this vaccination strategy will save 639–664 more lives and reduce hospitalization numbers by 1563–1633 by the end of June 2021. In Massachusetts, with an ample supply of vaccines (1.8M vaccines distributed by March), this same approach translated to 1647–1766 fewer hospitalizations and 3444–3647 fewer deaths compared to a random strategy, and a total of 11,716–12,424 fewer hospitalizations and 6278–6618 fewer deaths compared to a scenario with no vaccine. Our current seroprevalence estimates indicate that when the distribution campaign is finished on 4 March 2021, the population-level immunity (combined natural and vaccine-induced) in Rhode Island will be 71.3*%* and in Massachusetts will be 61.1*%*.

**Fig. 7:**
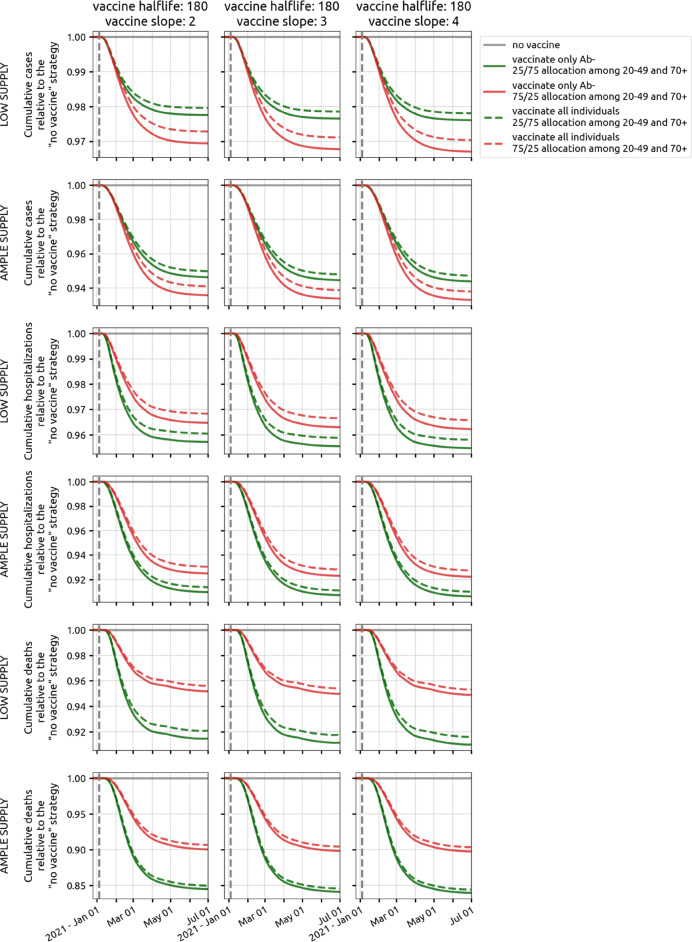
Difference between vaccinating all individuals (dashed lines) and vaccinating only antibody-negative individuals (solid lines). Three different vaccine profiles are considered (three columns). Plots show cumulative cases (rows 1 and 2), hospitalizations (rows 3 and 4), and deaths (rows 5 and 6) under both low supply (50,000 vaccines available) and ample supply (300,000 vaccines available). Green lines show the preferred 25/75 allocation in the 20–49 and 70+ age groups, and red lines show a 75/25 vaccine allocation. In Rhode Island, a vaccination campaign that is able to vaccinate only antibody-negative individuals will result in 0.5*%* to 1% fewer hospitalizations and deaths. Results for Massachusetts shown in Additional file 1: Figure S24

Vaccination of seropositive individuals can lead to wastage of vaccines, especially if the seropositive individuals were infected recently. Figure 7 shows that a policy of vaccinating only seronegative individuals would result in 0.5% to 1% fewer hospitalizations and deaths through mid-2021 than policy where serostatus is not checked prior to vaccination. Because the fraction of seropositive individuals in December/January was still low, a focus on vaccinating sero-negative individuals early in the vaccination campaign is not associated with a major public health benefit.

## Discussion

Two key characteristics of epidemic management that we account for when devising optimal vaccine distributions are (1) current seroprevalence levels in Rhode Island and Massachusetts and (2) the waning effect of vaccine efficacy. End-of-2020 attack rate was estimated to be 25.9% (95% CredInt: 24.5–27.2%) for Rhode Island and 20.6% (95% CredInt: 19.0–22.8%) for Massachusetts [29, 30]. These levels were too low to hope that vaccination campaigns would push populations past the point of herd immunity in early 2021. Nevertheless, assuming vaccine supplies would reach 20% to 30% coverage statewide by spring, both Rhode Island and Massachusetts would be able to reach approximately 60% to 70% population immunity by spring 2021, substantially slowing the spread of the virus by late spring and summer. These approximations assume our medium transmission scenario, and do not yet account for the arrival and spread of the higher-transmission B.1.1.7 lineage identified in the UK in late December [31, 32]. Vaccine efficacy is unlikely to wane so quickly that large groups of vaccinated individuals will be at risk of reinfection in mid-2021, however a small proportion of individuals infected in March-August 2020 may be at risk of reinfection if their antibody levels were to wane to sub-protective levels one year post-infection. The key variable to keep track of in summer/fall 2021 will be duration of immunity—both natural and vaccine-induced—to understand if the population-level risk of renewed outbreaks is likely to return in fall 2021. If duration of immunity is short, especially in the older age groups, booster vaccinations may be needed in late 2021.

The basic outcome observed in our evaluations is that high-mortality groups need to be vaccinated first in order to minimize death counts. In our model, groups that are at high risk of death if infected are the older age groups, but our analysis implies that any high-risk group—whether the risk factor is age, obesity, diabetes, past lung disease, lack of health care access, or anything else—should have equally high priority to vaccination. The obvious second-order implication is that individuals who have high-contact rates with risk groups or cohabit with someone at elevated risk of death should similarly be prioritized for vaccination. Although the highly specific context in which we have been managing COVID-19 risk over the past year allows us to identify such individuals on a case by case basis, there does not seem to be a systematic way to define this second-order group who have either frequent contacts or cohabitation with high-risk individuals; the clear exceptions are health care workers and employees of long-term care facilities.

Vaccination of high-contact groups alone is not an optimal approach, primarily due to the large mortality differences between the youngest and oldest age groups. This conclusion is identical to the one reached in Bubar et al. [15] and Moore et al. [33], but it differs from many common optimal vaccine allocations for influenza virus where distribution to high-contact groups can be optimal under a wide range of conditions [12–14]. For SARS-CoV-2 vaccine rollout optimization, both Hogan et al. [34] and Matrajt et al. [35] found that under some conditions, vaccination of younger age groups was optimal. Matrajt et al. [35] found that if vaccine supply is sufficient to cover 40% to 70% of the population and the vaccine campaign is modeled as instantaneous, then vaccination prioritization of younger age groups minimizes deaths. However, if the vaccine rollout is gradual, vaccination of the elderly is the optimal way to minimize deaths during the epidemic (their Figure 9). Likewise, Hogan et al. [34] found that under an intermediate level of vaccine supply (20% to 40%, their Figure 4) vaccination of children was optimal, but these modeling exercises assumed that children under 16 were eligible for vaccination and that the level of population immunity was around 11% to 16% at the time of vaccine rollout. Currently, there does not appear to be a general use-case where distribution of SARS-CoV-2 vaccines to younger individuals before older individuals results in fewer deaths during the course of a concurrent vaccination campaign and SARS-CoV-2 epidemic.

One efficiency that is important to evaluate in the early part of a vaccination campaign—especially when supply is moderate—is the deprioritization of vaccination for individuals with past confirmed COVID-19 infections or recent confirmed COVID-19 infections. Although this would normally be voluntary, public health communication around this topic could ask individuals with known past infection to offer up their place in the vaccination queue to those that are still fully susceptible. In Rhode Island and Massachusetts, more than 75% of symptomatic COVID-19 cases were likely identified through testing during 2020, meaning that a large majority of individuals would be aware that they had a past infection. Asymptomatically infected individuals would not know that they were COVID-positive at some point in 2020, unless they were included in a random screening campaign focused on nursing home staff/residents or essential workers. Most asymptomatic infections during 2020 would have occurred in the <20 or <30 age groups, individuals who would not be prioritized for vaccination, thus reducing the effect that serostatus would have on vaccine wastage in the early part of the campaign. Despite the good intentions of such a policy, our analysis shows that serological testing prior to vaccination would not provide a meaningful benefit to the campaign overall, and it would likely add to delays unless rapid high-specificity point-of-care IgG tests were used. Our model currently assumes that individuals with past known infection are not included in the winter/spring vaccination campaign; if these individuals are included, the hospitalization and mortality benefits of the vaccination campaign are predicted to be lower by about 0.5% to 1%.

The major benefit in focusing on state-level analyses of vaccine rollout is that cumulative seroprevalence and vaccination numbers can be tracked on a month by month basis. This is critical for public communication in the initial months of a vaccination campaign as the public will be eager to know whether total infection and vaccination counts are in the range of “30+10” or “40+20”, allowing the public health system to describe risk in terms of the fraction of individuals that are still not immune. Assuming our medium transmission scenario through spring and improved vaccination trends at approximately 0.5% of the population per day, both Massachusetts and Rhode Island were predicted [36] to cross the 50% immune mark in February or March 2021, and it is likely that this occurred according to inference done in early 2021 [29, 37]. As the more transmissible B.1.1.7 lineage began circulating in New England at higher levels in spring 2021—with a third to half of sequenced viruses identified as B.1.1.7 [38]—it is imperative in spring and summer to be cautious on the re-starting of larger group activities until a substantial majority of each state’s population is vaccinated. This new variant is associated with a higher herd-immunity threshold, and re-opening too quickly or too early would risk generating new outbreaks while the vaccination campaign is not yet complete.

### Limitations

Our modeling approach has several limitations. First, although age stratification allows for a straightforward strategy design focused on protecting the elderly, our model does not include any variables on race, comorbidities, health care workers, or other essential workers. This means that some key high-contact and high-risk groups are omitted from the modeling, and it is unlikely that an age-group proxy would be a suitable substitute for any of them. For example, health care workers would likely fall into the high-contact category, but they may preferentially be in contact with non-susceptibles (i.e., SARS-CoV-2 positives) making them more a high-exposure group than a high-contact group. In addition, the benefits of vaccinating HCWs and essential workers is that certain essential services (hospitals, schools, grocery stores) can continue functioning, a benefit not captured in traditional epidemiological models.

Second, phase 3 efficacy trials of the Pfizer/BioNTech and Moderna vaccines were not designed to evaluate reductions in transmission. Thus it is not possible to state whether the vaccines’ high efficacy could be compromised by asymptomatic or sub-clinical infections occurring in vaccinated individuals and allowing for the continuation of transmission. Asymptomatic individuals do have lower viral loads and fewer opportunities to transmit via large droplets projected out through coughs, sneezes, speech, or breathing. Therefore, inadequate vaccine prevention of potentially-transmissible asymptomatic infections may reduce the indirect benefits of vaccination, but this effect size is currently unknown (and possibly small). Asymptomatic and pre-symptomatic infection does occur for SARS-CoV-2, but the naturally observed infectivities in household and contact tracing contexts are likely to be different than the infectivity of a vaccinated infected asymptomatic individual. Preliminary reports from a Moderna FDA filing [39] were interpreted as early evidence that the Moderna vaccine may offer some degree of protection against asymptomatic infection. Three large cohorts that reported results in 2021 showed that vaccine efficacy against infection is above 70% [40–42] with differing efficacy against variants [41]. The efficacy against transmission is not known, but the efficacy of a vaccine against transmission given that a vaccinee is infected has been estimated at 43% (Pfizer/BioNTech) and 45% (AstraZeneca) [43]. In our model analysis, if vaccinated individuals were added to a “partially susceptible pool” and allowed to be infected with mild or no clinical symptoms, case numbers would increase but the hospitalization and death outcomes in our analysis are unlikely to be affected.

Third, results on long-term efficacy and efficacy by age are currently unknown. The combination of these two is critical as older individuals may be protected for a shorter time than younger healthier individuals. If vaccine-induced immunity were to wane quickly in older individuals, the vaccine would be least effective in the group that needs it the most. All vaccination campaigns would then need to be restructured as routine/repeat campaigns that focus on the most vulnerable individuals and account for the fact that re-vaccination may need to occur often to realize the vaccination campaign’s intended mortality benefits.

## Conclusions

Real-time knowledge of seroprevalence can guide decisions on vaccine allocation. Knowing which population groups have experienced the most infection and how far seroprevalence has advanced population-wide can help determine the total vaccine supply needed as well as its distribution priorities. This highlights the value of (1) real-time attack-rate estimation, (2) completeness of population surveillance, and (3) widespread testing. Comprehensive surveillance and widespread testing ensure that individuals are as informed as possible about their serostatus to SARS-CoV-2 and allows for the health system to communicate to seropositive individuals that they should forego vaccination in the first few rounds of allocation while immunologically naive individuals are vaccinated first. Given the 1 January 2021 seroprevalence levels in Rhode Island and Massachusetts, older age groups and vulnerable populations with substantial risk of hospitalization or death resulting from SARS-CoV-2 infection should be the priority groups for vaccination, after the completion of vaccine rollout for front-line health and medical workers.

## Supplementary Information

**Additional file 1** Supplementary figures.

## Data Availability

All data and code are available at https://github.com/bonilab/covid19-vaccine-allocation-RI-MA.

## Abbreviations

BE: Belgium
COVID or COVID-19: Coronavirus disease 2019
CredInt: Credible interval
FOI: Force of infection
HCW: Health care worker
IQR: Interquartile range
MA: Massachusetts
PCR: Polymerase chain reaction
RI: Rhode Island
UK: United Kingdom
VE: Vaccine efficacy

## References

[1] Holmes EC, Zhang Y-Z. Novel 2019 coronavirus genome. 2020. https://virological.org/t/novel-2019-coronavirus-genome/319. Accessed 11 Dec 2020.

[2] Cohen J (2020). Vaccine designers take first shots at COVID-19. Science.

[3] New York Times. Coronavirus vaccine tracker. 2020. https://www.nytimes.com/interactive/2020/science/coronavirus-vaccine-tracker.html. Accessed 15 Dec 2020.

[4] US Food and Drug Administration. Pfizer-BioNTech COVID-19 Vaccine. 2020. https://www.fda.gov/emergency-preparedness-and-response/coronavirus-disease-2019-covid-19/pfizer-biontech-covid-19-vaccine. Accessed 15 Dec 2020.

[5] US Food and Drug Administration. Moderna COVID-19 Vaccine. 2020. https://www.fda.gov/emergency-preparedness-and-response/coronavirus-disease-2019-covid-19/moderna-covid-19-vaccine. Accessed 30 Dec 2020.

[6] US Food and Drug Administration. FDA Issues Emergency Use Authorization for Third COVID-19 Vaccine. 2021. https://www.fda.gov/news-events/press-announcements/fda-issues-emergency-use-authorization-third-covid-19-vaccine. Accessed 13 May 2021.

[7] Dooling K. The Advisory Committee on Immunization Practices’ Interim Recommendation for Allocating Initial Supplies of COVID-19 Vaccine — United States, 2020. MMWR Morb Mortal Wkly Rep. 2020; 69. 10.15585/mmwr.mm6949e1. 10.15585/mmwr.mm6949e1 PMC7737687 33301429

[8] McClung N, Chamberland M, Kinlaw K, Matthew DB, Wallace M, Bell BP, Lee GM, Talbot HK, Romero JR, Oliver SE, Dooling K. The Advisory Committee on Immunization Practices’ Ethical Principles for Allocating Initial Supplies of COVID-19 Vaccine — United States, 2020. MMWR Morb Mortal Wkly Rep. 2020; 69. URL 10.15585/mmwr.mm6947e3. 10.15585/mmwr.mm6947e3 PMC7727606 33237895

[9] Dooling K. Phased Allocation of COVID-19 Vaccines. COVID-19 Vaccines Work Group, ACIP. 2020. https://www.cdc.gov/vaccines/acip/meetings/downloads/slides-2020-12/COVID-02-Dooling.pdf. Accessed 15 Dec 2020.

[10] Rhode Island Department of Health. COVID-19 Vaccination Plan (Interim Draft), 16OCT2020. 2020. https://health.ri.gov/publications/plans/RI-COVID-19-Vaccination-Plan-Interim-Draft.pdf. Accessed 15 Dec 2020.

[11] Massachusetts Department of Public Health. COVID-19 Vaccine Presentation, Baker-Polito Administration, December 9, 2020. 2020. https://www.mass.gov/doc/ma-covid-19-vaccine-presentation-1292020/download. Accessed 15 Dec 2020.

[12] Bansal S, Pourbohloul B, Meyers LA (2006). A comparative analysis of influenza vaccination programs. PLoS Med.

[13] Dushoff J, Plotkin JB, Viboud C, Simonsen L, Miller M, Loeb M, Earn DJD (2007). Vaccinating to protect a vulnerable subpopulation. PLoS Med.

[14] Medlock J, Galvani AP (2009). Optimizing Influenza Vaccine Distribution. Science.

[15] Bubar KM, Reinholt K, Kissler SM, Lipsitch M, Cobey S, Grad YH, Larremore DB. Model-informed covid-19 vaccine prioritization strategies by age and serostatus. medRxiv. 2020. 10.1101/2020.09.08.20190629. 10.1126/science.abe6959 PMC7963218 33479118

[16] Reichert TA, Sugaya N, Fedson DS, Glezen WP, Simonsen L, Tashiro M (2001). The Japanese experience with vaccinating schoolchildren against influenza. N Engl J Med.

[17] Levin AT, Hanage WP, Owusu-Boaitey N, Cochran KB, Walsh SP, Meyerowitz-Katz G. Assessing the age specificity of infection fatality rates for COVID-19: systematic review, meta-analysis, and public policy implications. Eur J Epidemiol. 2020. 10.1007/s10654-020-00698-1. 10.1007/s10654-020-00698-1 PMC7721859 33289900

[18] Wikle NB, Tran TN-A, Gentilesco B, Leighow SM, Albert J, Strong ER, Břinda K, Inam H, Yang F, Hossain S, Chan P, Hanage WP, Messick M, Pritchard JR, Hanks EM, Boni MF. SARS-CoV-2 epidemic after social and economic reopening in three US states reveals shifts in age structure and clinical characteristics. medRxiv. 2020:2020.11.17.20232918. 10.1101/2020.11.17.20232918. 10.1126/sciadv.abf9868 PMC8791616 35080987

[19] Andreasen V (2003). Dynamics of annual influenza A epidemics with immuno-selection. J Math Biol.

[20] Boni MF, Gog JR, Andreasen V, Christiansen FB (2004). Influenza drift and epidemic size: the race between generating and escaping immunity. Theor Popul Biol.

[21] Andreasen V (2011). The Final Size of an Epidemic and Its Relation to the Basic Reproduction Number. Bull Math Biol.

[22] Penn State CIDD COVID Modeling Team. Attack rates through November 30. 2020. https://mol.ax/covid/attack-rate-12-15/. Accessed 15 Dec 2020.

[23] Coletti P, Wambua J, Gimma A, Willem L, Vercruysse S, Vanhoutte B, Jarvis CI, Van Zandvoort K, Edmunds J, Beutels P, Hens N (2020). CoMix: comparing mixing patterns in the Belgian population during and after lockdown. Sci Rep.

[24] Funk S. Socialmixr: Social Mixing Matrices for Infectious Disease Modelling. 2020. R package version 0.1.7. https://CRAN.R-project.org/package=socialmixr.

[25] Jarvis CI, Van Zandvoort K, Gimma A, Prem K, Auzenbergs M, O’Reilly K, Medley G, Emery JC, Houben RMGJ, Davies N, Nightingale ES, Flasche S, Jombart T, Hellewell J, Abbott S, Munday JD, Bosse NI, Funk S, Sun F, Endo A, Rosello A, Procter SR, Kucharski AJ, Russell TW, Knight G, Gibbs H, Leclerc Q, Quilty BJ, Diamond C, Liu Y, Jit M, Clifford S, Pearson CAB, Eggo RM, Deol AK, Klepac P, Rubin GJ, Edmunds WJ, CMMID COVID-19 working group (2020). Quantifying the impact of physical distance measures on the transmission of COVID-19 in the UK. BMC Med.

[26] ClinicalTrials.gov [Internet]. Bethesda (MD): National Library of Medicine (US). Identifier NCT04368728, Study to describe the safety, tolerability, immunogenicity, and efficacy of RNA vaccine candidates against COVID-19 in healthy individuals. 2020. https://www.clinicaltrials.gov/ct2/show/NCT04368728. Accessed 31 Dec 2020.

[27] ClinicalTrials.gov [Internet]. Bethesda (MD): National Library of Medicine (US). Identifier NCT04470427, A study to evaluate efficacy, safety, and immunogenicity of mRNA-1273 vaccine in adults aged 18 years and older to prevent COVID-19. 2020. https://clinicaltrials.gov/ct2/show/NCT04470427. Accessed 31 Dec 2020.

[28] Davies NG, Klepac P, Liu Y, Prem K, Jit M, Eggo RM (2020). Age-dependent effects in the transmission and control of COVID-19 epidemics. Nat Med.

[29] Penn State COVID-19 Modeling Collaboration. https://twitter.com/maciekboni/status/1362257842934317059. Accessed 17 Feb 2021.

[30] Penn State COVID-19 Modeling Collaboration. https://twitter.com/maciekboni/status/1351904374994444288. Accessed 20 Jan 2021.

[31] Rambaut A, Loman N, Pybus O, Barclay W, Barrett J, Carabelli A, Connor T, Peacock T, Robertson DL, Volz E, COVID19GenomicsConsortiumUK(CoG-UK). Preliminary genomic characterisation of an emergent SARS-CoV-2 lineage in the UK defined by a novel set of spike mutations. 2020. https://virological.org/t/preliminary-genomic-characterisation-of-an-emergent-sars-cov-2-lineage-in-the-uk-defined-by-a-novel-set-of-spike-mutations/563. Accessed 20 Dec 2020.

[32] Volz E, Mishra S, Chand M, Barrett JC, Johnson R, Hinsley WR, Laydon DJ, Dabrera G, O’Toole A, Ragonnet-Cronin M, Loman NJ, McCrone JT, Gonçalves S, Jorgensen D, Hill V, Jackson DK, Gaythorpe K, Groves N, Sillitoe J, Kwiatkowski P, Bhatt S, Hopkins S, Gandy A, Rambaut A, Ferguson NM. Transmission of SARS-CoV-2 Lineage B.1.1.7 in England: Insights from linking epidemiological and genetic data. MRC Centre for Global Infectious Disease Analysis (Report 42). https://www.imperial.ac.uk/mrc-global-infectious-disease-analysis/covid-19/report-42-sars-cov-2-variant/. Accessed: 3 Jan 2021.

[33] Moore S, Hill EM, Dyson L, Tildesley MJ, Keeling MJ (2021). Modelling optimal vaccination strategy for SARS-CoV-2 in the UK. PLOS Comput Biol.

[34] Hogan AB, Winskill P, Watson OJ, Walker PGT, Whittaker C, Baguelin M, Brazeau NF, Charles GD, Gaythorpe KAM, Hamlet A, Knock E, Laydon DJ, Lees JA, Løchen A, Verity R, Whittles LK, Muhib F, Hauck K, Ferguson NM, Ghani AC. Within-country age-based prioritisation, global allocation, and public health impact of a vaccine against SARS-CoV-2: A mathematical modelling analysis. Vaccine. 2021. 10.1016/j.vaccine.2021.04.002. 10.1016/j.vaccine.2021.04.002 PMC8030738 33933313

[35] Matrajt L, Eaton J, Leung T, Brown ER (2021). Vaccine optimization for COVID-19: Who to vaccinate first?. Sci Adv.

[36] Tran TN-A, Wikle NB, Albert E, Inam H, Strong E, Brinda K, Leighow SM, Yang F, Hossain S, Pritchard JR, Chan P, Hanage WP, Hanks EM, Boni MF. Optimal sars-cov-2 vaccine allocation using real-time seroprevalence estimates in rhode island and massachusetts. medRxiv. 2021. 10.1101/2021.01.12.21249694. 10.1186/s12916-021-02038-w PMC8275456 34253200

[37] Penn State COVID-19 Modeling Collaboration. https://twitter.com/maciekboni/status/1373081146423189505. Accessed 19 Mar 2021.

[38] Latif AA, Mullen JL, Alkuzweny M, Tsueng G, Cano M, Haag E, Zhou J, Zeller M, Matteson N, Wu C, Andersen KG, Su AI, Gangavarapu K, Hughes LD, and the Center for Viral Systems Biology. outbreak.info. https://outbreak.info/location-reports?loc=USA. Accessed 13 May 2021.

[39] US Food and Drug Administration. Vaccines and Related Biological Products Advisory Committee Meeting Presentation. 2020. https://www.fda.gov/media/144453/download. Accessed 31 Dec 2020.

[40] Hall VJ, Foulkes S, Saei A, Andrews N, Oguti B, Charlett A, Wellington E, Stowe J, Gillson N, Atti A, Islam J, Karagiannis I, Munro K, Khawam J, Group TSS, Chand MA, Brown C, Ramsay ME, Bernal JL, Hopkins S (2021). Effectiveness of BNT162b2 mRNA vaccine against infection and COVID-19 vaccine coverage in healthcare workers in England, multicentre prospective cohort study (the SIREN study). SSRN Pre-print.

[41] Abu-Raddad LJ, Chemaitelly H, Butt AA. Effectiveness of the BNT162b2 Covid-19 Vaccine against the B.1.1.7 and B.1.351 Variants. N Engl J Med. 2021; 0(0). 10.1056/NEJMc2104974. 10.1056/NEJMc2104974 PMC8117967 33951357

[42] Pritchard E, Matthews PC, Stoesser N, Eyre DW, Gethings O, Vihta K-D, Jones J, House T, VanSteenHouse H, Bell I, Bell JI, Newton JN, Farrar J, Diamond I, Rourke E, Studley R, Crook D, Peto T, Walker AS, Pouwels KB. Impact of vaccination on sars-cov-2 cases in the community: a population-based study using the uk’s covid-19 infection survey. medRxiv. 2021. 10.1101/2021.04.22.21255913.

[43] Harris RJ, Hall JA, Zaidi A, Andrews NJ, Dunbar JK, Dabrera G (2021). Impact of vaccination on household transmission of sars-cov-2 in england.

